# Overexpression of a Cinnamyl Alcohol Dehydrogenase-Coding Gene, *GsCAD1*, from Wild Soybean Enhances Resistance to Soybean Mosaic Virus

**DOI:** 10.3390/ijms232315206

**Published:** 2022-12-02

**Authors:** Hongwei Xun, Xueyan Qian, Meng Wang, Jiaxin Yu, Xue Zhang, Jinsong Pang, Shucai Wang, Lili Jiang, Yingshan Dong, Bao Liu

**Affiliations:** 1 Key Laboratory of Molecular Epigenetics of MOE, Northeast Normal University, Changchun 130024, China; 2 Jilin Provincial Key Laboratory of Agricultural Biotechnology, Jilin Academy of Agricultural Sciences, Changchun 130033, China

**Keywords:** *GsCAD1*, wild soybean, soybean mosaic virus, salicylic acid, lignin

## Abstract

Soybean mosaic virus (SMV) is the most prevalent soybean viral disease in the world. As a critical enzyme in the secondary metabolism of plants, especially in lignin synthesis, cinnamyl alcohol dehydrogenase (CAD) is widely involved in plant growth and development, and in defense against pathogen infestation. Here, we performed RNAseq-based transcriptome analyses of a highly SMV-resistant accession (BYO-15) of wild soybean (*Glycine soja*) and a SMV-susceptible soybean cultivar (Williams 82), also sequenced together with a resistant plant and a susceptible plant of their hybrid descendants at the F3 generation at 7 and 14 days post-inoculation with SMV. We found that the expression of *GsCAD1* (from *G. soja*) was significantly up-regulated in the wild soybean and the resistant F3 plant, while the *GmCAD1* from the cultivated soybean (*G. max*) did not show a significant and persistent induction in the soybean cultivar and the susceptible F3 plant, suggesting that *GsCAD1* might play an important role in SMV resistance. We cloned *GsCAD1* and overexpressed it in the SMV-susceptible cultivar Williams 82, and we found that two independent *GsCAD1*-overexpression (OE) lines showed significantly enhanced SMV resistance compared with the non-transformed wild-type (WT) control. Intriguingly, the lignin contents in both OE lines were higher than the WT control. Further liquid chromatography (HPLC) analysis showed that the contents of salicylic acid (SA) were significantly more improved in the OE lines than that of the wild-type (WT), coinciding with the up-regulated expression of an SA marker gene. Finally, we observed that *GsCAD1*-overexpression affected the accumulation of SMV in leaves. Collectively, our results suggest that *GsCAD1* enhances resistance to SMV in soybeans, most likely by affecting the contents of lignin and SA.

## 1. Introduction

The soybean (*Glycine max* (L.) Merr.) is a major source of vegetal protein and oil for human and domestic animals [1]. Soybean mosaic virus (SMV) is one of the most prevalent soybean viral diseases world-wide [2]. SMV causes about 35–50% yield loss annually [3]. The most cost-effective way to mitigate the SMV-incurred yield loss of soybeans is to breed resistant cultivars [4].

SMV was usually divided into seven strains, namely, G1–G7 [5,6], while in China, SMV was divided into 21 strains, namely, SMV-SC1 through SMV-SC21 [7,8]. Three resistance loci (*Rsv1*, *Rsv3* and *Rsv4*) have been identified in soybeans, which are located in chromosomes 13, 14 and 2, respectively [9,10,11]. The *Rsv1* locus renders plants resistant to SMV strains G1–G3, but susceptible to strains G5–G7 [12]. The *Rsv3* locus confers resistance to SMV strains G5–G7, but not to G1–G4 [13]. The *Rsv4* locus can resist all the SMV strains at the seedling stage but often produces systemic symptoms in mature plants [14]. To date, several anti-SMV genes have been cloned, including *GmPEX14* [15], *GmAKT2* [16], *GmPP2C3a* [17], *GmCnx1* [18], *GmeEF1a* [19], *GmSN1* [20], *GmKR3* [21], *GmCYB5* [22], *GmF3H1, GmF3H2* and *GmFNSII-1* [23], *GmST1* [24], *Rsc4-3* [25], *GmPAP2.1* [26] and *GmCAL* [27]. The functional mechanisms of these anti-SMV genes are mainly through regulating immune responses via increasing the contents of phytohormones (ABA or SA) or secondary metabolites (e.g., isoflavone) or *R* genes’ recognition of viral-encoded protein to interfere with the replication or movement of SMV in the soybean host cells.

Lignin is an important aromatic polymer derived from the oxidative polymerization of hydroxycinnamyl alcohol [28]. In tracheophytes, lignin is associated with plant growth and enhances resistance to environment stresses [29]. The genes involved in lignin biosynthesis were activated during plant–pathogen interactions [30,31]. However, the deposition of lignin as a physical barrier can withstand the expending of pathogens [32]. Cinnamyl alcohol dehydrogenase (CAD) is an important enzyme for lignin biosynthesis, which catalyzes the reduction of cinnamaldehydes into cinnamyl alcohol, the last step of monolignol biosynthesis before polymerization [33]. CAD is encoded by a multigene family and has been found in many species including plants and bacteria [34]. In gymnosperm, CAD has high catalytic activity for coniferaldehyde and a low affinity for sinapaldehyde; in contrast, CAD from angiosperm has a high affinity to both substrates [35]. In *Arabidopsis thaliana*, there are nine CAD proteins, which can be categorized into four classes based on their peptide similarities [33]. In *A. thaliana*, *CAD-C* and *CAD-D* are the main lignin synthesis genes with partially redundant functions. The double mutants of *cad-c* and *cad-d* reduced resistance to *Pseudomonas syringae* pv. Tomato [32]. Additionally, it has been found that the down-regulation of the *CAD-C* gene could impair plant growth and defense [36]. Overexpression of *CAD12* significantly increased the resistance to *Rhizoctonia cerealis* in wheat [37]. There are in total 12 *CAD* and *CAD*-like genes in rice, of which *OsCAD2* had the highest enzymatic activity, and *OsCAD2* and *OsCAD6* were up-regulated by *Xanthomonas oryzae* pv. oryzae infection and UV irradiation, respectively [38]. *Rhizoctonia solani* is a widespread plant fungus that can cause sheath blight in maize and rice, and an F-box protein, ZmFBL41, that has two amino acid substitutions in this allele was found to prevent its interaction with ZmCAD, thereby avoiding the ubiquitination and degradation of ZmCAD, and hence, promoted the accumulation of lignin and improved resistance [39].

The wild soybean (*Glycine soja* Sieb. and Zucc.) is the direct progenitor of the cultivated soybean (*G. max*), which has rich genetic diversity and contains many useful genes conferring resistance/tolerance to pathogens and abiotic stresses [40]. Discovering beneficial genes from wild soybeans and transferring them into cultivated soybeans is an effective way to breed new soybean varieties. For example, *GsAAE3* was cloned from wild soybean accession BW69 and its overexpression was found to enhance tolerance to cadmium and aluminum in *Arabidopsis* and soybean hairy roots [41]. Thirty-four salt-tolerant varieties were identified from wild soybean germplasm resources, based on genome-wide association analysis combined with gene expression; it was found that a 7 bp insertion/deletion of *GsERD15B* was significantly associated with salt tolerance; overexpression of *GsERD15B* could enhance the salt tolerance of soybeans by increasing the expression level of abscisic acid signal transduction, proline content and related genes [42]. In soybeans, GsBET11a, the C-terminal transmembrane domain of TMD, can interact with GsCBRLK to mediate the subcellular localization of GsCRCK1s to regulate salt stress response [43]. GsRSS3L, encoding a RICE SALT SENSITIVE 3-like protein from wild soybean ZYD00006 (resistance to seed coat mottling), can inhibit the multiplication of SMV in *Nicotiana benthamiana* [44].

Here, we performed transcriptome profiling to analyze gene expression post-SMV-inoculation in a highly resistant wild soybean accession (BYO-15) and a susceptible soybean cultivar Williams 82 and their hybrid descendants in the F3 generation. We found that overexpression of *GsCAD1* from wild soybeans significantly enhanced SMV resistance in Williams 82, which was most likely due to elevating the content of lignin and SA.

## 2. Results

### 2.1. Identification of GsCAD1 as a Possible anti-SMV Gene

**Figure 1:**
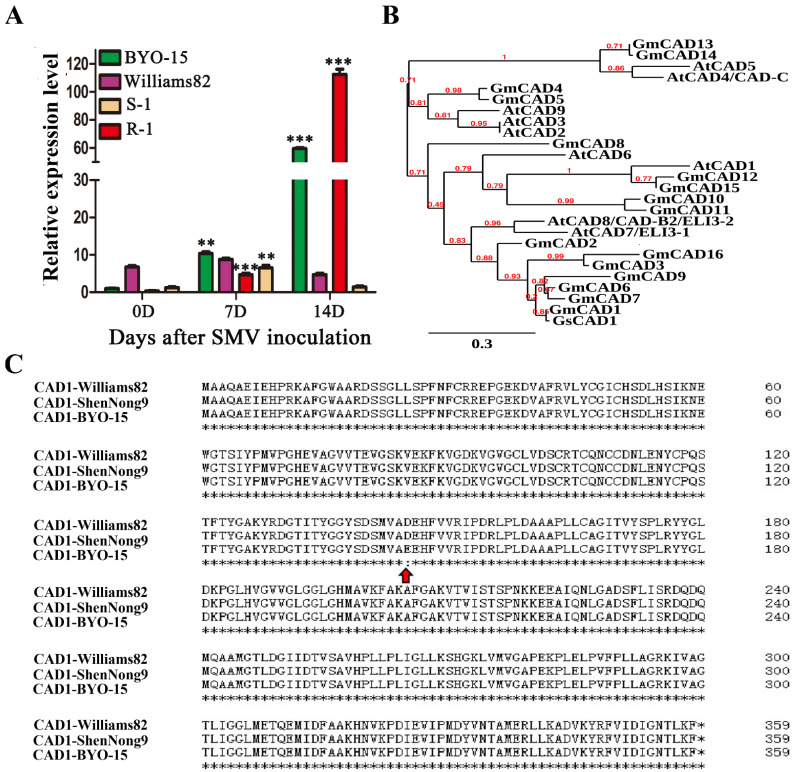
Mining anti-SMV gene of *GsCAD1*. (**A**) Expression changes of *CAD1* gene in highly resistant wild soybeans and susceptible cultivar and their hybrid descendants in S3 generation including one resistant plant (R-1) and one susceptible plant (S-1) before and after SMV inoculation based on qRT-PCR (*n* = 3), and the asterisks represent significant differences of expression between wild and cultivar soybeans (Student’s *t*-test, **, *p* < 0.01; ***, *p* < 0.001). (**B**) Neighbor-join (NJ) phylogeny tree of 16 CAD members in soybean from the reference genome of Williams 82. The percentage represents the supported ratio based on 100 bootstraps. (**C**) Amino acid alignments of CAD1 among Willianms82, shennong9 and BYO-15. The red arrow represents the missense mutation site on the 145 amino acids (E145D), between the wild soybean (BYO-15) and the SMV-susceptible soybean cultivars (Williams 82 and Shennong 9).

The wild soybean (*G. soja*) has a rich genetic diversity and contains many desirable genes for disease resistance in cultivated soybeans [40]. To clone anti-SMV genes from wild soybeans, RNA-Seq was used to analyze the gene expression changes of a SMV-resistant wild soybean accession BYO-15, a susceptible soybean cultivar Williams 82 and their hybrid descendants in the F3 generation (including one resistant plant R-1 and one susceptible plant S-1) (Figure S1 and Tables S1–S8). The first criterion for candidate gene selection is genes whose expression is persistently up-regulated in both the SMV-resistant wild soybean accession BYO-15 and R-1 (log2 (fold change) ≥ 2) due to the SMV induction. We identified 110 candidate genes meeting this criterion (Figure S2A). Next, we interrogated the expression of these 110 genes in Williams 82 (the susceptible cultivar) and plant S-1; we found that many genes showed similar expression induction patterns in one or both of these susceptible plant types. Furthermore, we found that the homolog of *GsCAD1* in cultivated soybean cv. Williams 82, *GmCAD1*, did not show induced expression at 7 days and 14 days after SMV induction and even showed down-regulation at 14 days after SMV induction in Williams 82 and S-1 (Figure S2B). Consequently, we suspected that *GsCAD1* (from wild soybean BYO-15) was likely a candidate anti-SMV gene. The contrasted expression patterns of *GsCAD1* in BYO-15 and R-1 vs. *GmCAD1* in Williams 82 and S-1 were verified by qRT-PCR (Figure 1A). We thus speculated that *GsCAD1* may play an important role in SMV resistance. We identified that there were 16 CAD-like proteins in Williams 82 according to the conserved domain and “GLGGLG” motif on the basis of CAD peptides alignment from *A. thaliana* and soybeans. We could also distinguish between the different CAD genes from the phylogenetic tree, and found that *GsCAD1* and *GmCAD1* are closely related to *AtCAD7* and *AtCAD8* of *A. thaliana* (Figure 1B). There was a single nucleotide polymorphism (SNP) (T435A) between *GsCAD1* and *GmCAD1*, which resulted in a missense mutation, i.e., glutamic acid in wild soybeans and aspartic acid in cultivated soybeans (E145 D) (Figure 1C). This missense mutation is in an alcohol dehydrogenase GroES-like domain. We also performed protein secondary structure prediction and found that the existence of this SNP indeed affects the protein secondary structure, which may enable a change from a random coil (GmCAD1) to a β-sheet (GsCAD1) at the 145 amino acid (Figure S3) [45], potentiating their functional difference. We thus chose *GsCAD1* as a potential SMV-resistant candidate gene for further study.

### 2.2. Overexpression of GsCAD1 in Soybean Increased Resistance to SMV

**Figure 2:**
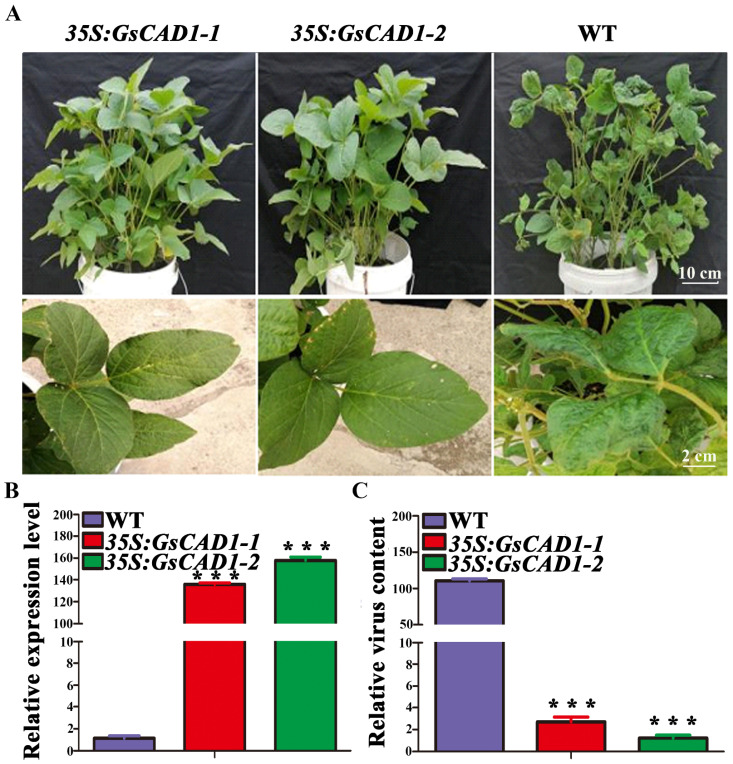
Overexpression of *GsCAD1* in soybeans increased resistance to SMV. (**A**) Phenotypes of the wild-type and two *35S:GsCAD1* transgenic plant lines after SMV inoculation. The phenotypes of pot-grown *35S:GsCAD1* transgenic and the wild-type plants after SMV inoculation (the top three panels) and the close view of the newly expanded unifoliate leaves (the bottom three panels) are shown. (**B**) Relative expression levels of *GsCAD1* in the wild-type and *35S:GsCAD1* transgenic plants at 35 days of SMV inoculation. Data represent the mean ± SD of three independent experiments, with each experiment consisting of three technical replicates. The asterisks represent significantly different expression levels between WT and OE lines (Student’s *t*-test, ***, *p* < 0.001). (**C**) Virus content in the wild-type and *35S:GsCAD1* transgenic plants (*CP* gene). Data represent the mean ± SD of three independent experiments at 35 days of SMV inoculation, with each experiment consisting of three technical replicates. The asterisks represent significantly different virus contents between WT and OE lines (Student’s *t*-test, ***, *p* < 0.001).

CAD is an important enzyme involved in lignin biosynthesis, which is known to play an important role in plant resistance to different pathogens [33,37,38,39,44]. In order to test whether *GsCAD1* may confer resistant to SMV, we obtained transgenic plants with an independent and stable inheritance of *GsCAD1* by using the soybean cotyledon node transformation method, and analyzed the expression level of *GsCAD1* in transgenic plants. We found that the expression level of *GsCAD1* in the transgenic plants was significantly higher than that of non-transgenic WT plants (Figure 2B). Through the SMV infection experiment, SMV-inoculated WT plants showed typical disease symptoms 30 days after inoculation, but transgenic plants did not. This indicated that the *GsCAD1*-overexpressing (OE) transgenic plants significantly improved resistance to SMV (Figure 2A). The virus content (*CP* gene) of the transgenic plants before and after inoculation with SMV was quantified. Results showed that the content of SMV in the transgenic plants was much lower than that of the WT plants (Figure 2C). These results indicated that overexpression of *GsCAD1* can improve the resistance to SMV in soybeans.

### 2.3. GsCAD1-Overexpressing Transgenic Soybean Lines Increased the Lignin Content before and after SMV Inoculation

**Figure 3:**
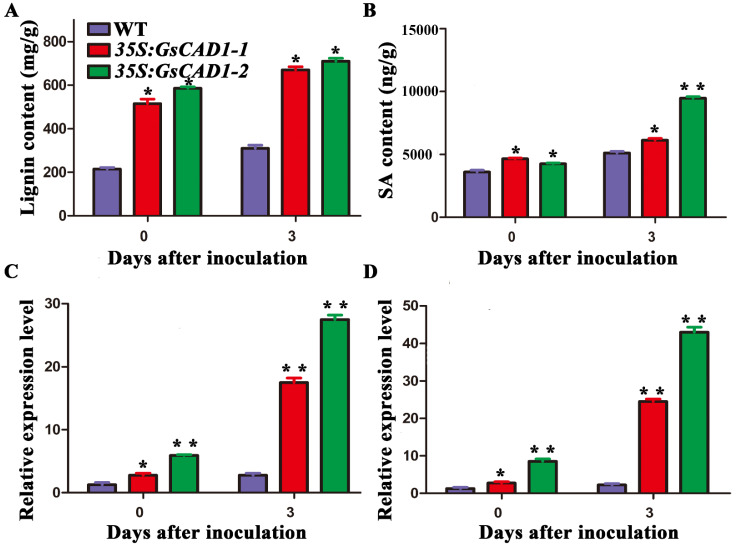
Increased lignin and SA content and expression levels of related genes in *GsCAD1*-overexpression transgenic soybean lines before and after SMV inoculation. (**A**) Changes of lignin content between the wild-type and *35S:GsCAD1* transgenic plants before and 3 days after inoculation with SMV, respectively. At 14 days, the wild-type and *35S:GsCAD1* transgenic plants were inoculated with virus. The inoculated leaflets were collected immediately before inoculation (0 h) and 3 days after the SMV inoculation. The asterisks in each panel represent significant content and/or expression differences between WT and OE lines (Student’s *t*-test, *, *p* < 0.05). (**B**) SA contents in the wild-type and *35S:GsCAD1* transgenic plants. At 14 days, the wild-type and *35S:GsCAD1* transgenic plants were inoculated with virus. The inoculated leaflets were collected immediately before inoculation (0 h) and 3 days after the SMV inoculation. Endogenous SA was measured by HPLC. Data represent the mean ± SD of three replicates. The asterisks in each panel represent significant content and/or expression differences between WT and OE lines (*, *p* < 0.05, **, *p* < 0.01). (**C**) Expression of SA synthetic gene *PAL-03g* in the wild-type and *35S:GsCAD1* transgenic plants before inoculation (0 h) and 3 days after the SMV inoculation. Data represent the mean ± SD of three independent experiments at 35 days of SMV inoculation, with each experiment consisting of three technical replicates. * Significantly different from that in the wild-type (*, *p* < 0.05, **, *p* < 0.01). (**D**) Expression of SA-responsive gene *PR1* in the wild-type and *35S:GsCAD1* transgenic plants before inoculation (0 h) and 3 days after the SMV inoculation. Data represent the mean ± SD of three independent experiments at 35 days of SMV inoculation, with each experiment consisting of three technical replicates. The asterisks in each panel represent significant content and/or expression differences between WT and OE lines (*, *p* < 0.05; **, *p* < 0.01).

Lignin is a kind of vital aromatic polymer, which is associated with plant growth and can act as a physical barrier to withstand the expending of pathogens [7,8,17,30]. Through gene function prediction, it was found that *GsCAD1* may play a key role in the synthesis of lignin. We generated two independent stable *GsCAD1*-OE transgenic soybean lines via selfing to the T3 generation, named *35S:GsCAD1-1* and *35S:GsCAD1-2*. We measured the lignin content in both the transgenic *GsCAD1*-OE lines and WT plants before and 3 days after SMV inoculation. The lignin contents in both transgenic *GsCAD1*-OE lines were significantly higher than that of non-transgenic WT plants (Figure 3A). This suggests that one mechanism for *GsCAD1*-conditioned SMV resistance is likely via increased lignin content.

### 2.4. GsCAD1-Overexpressing Transgenic Soybean Lines Showed Increased Salicylic Acid Contents before and after Inoculation with SMV

**Figure 4:**
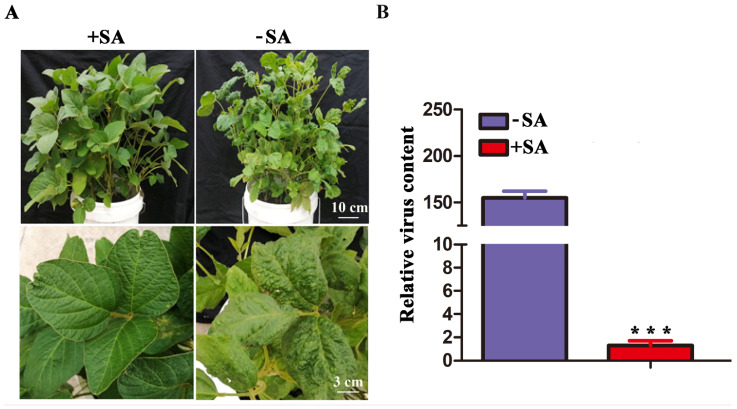
Spraying SA improved the resistance to SMV in soybeans. (**A**) Phenotypes of wild-type plants with (top left panel) and without (top right panel) SA treatment plants after SMV inoculation, respectively; close view of the newly expanded unifoliate leaves with (bottom left panel) and without (bottom right panel) were also shown. (**B**) Virus content in wild-type plants with and without SA treatment (*CP* gene). Data represent the mean ± SD of three independent experiments at 35 days of SMV inoculation, with each experiment consisting of three technical replicates. The asterisks represent significantly different virus contents (Student’s *t*-test, ***, *p* < 0.001).

Many studies reported that salicylic acid (SA) plays an important role in defense against the infection of plant pathogens [13,25,46]. We thus used HPLC to detect possible changes in SA in the *GsCAD1*-OE transgenic soybean lines relative to WT plants before and after inoculation with SMV. We found that the SA contents in the *GsCAD1*-OE transgenic soybean lines were significantly higher than those of the WT after SMV inoculation (Figure 3B). We also used qRT-PCR to detect the expression of key SA synthesis genes before and after SMV inoculation. We found that the expression levels of a SA synthesis gene *GmPAL-03g* (*Glyma03g33880*) and a SA-responsive gene *PR1* were significantly higher than those in the WT (Figure 3C,D); however, the expression level of *GmICS* and other *GmPALs* did not change between the transgenic lines and WT (data not shown). These results indicate that *GsCAD1*-overexpression promoted the synthesis of SA, which in turn may have contributed to the resistance to SMV. We further found that SA could indeed mitigate the amplification of SMV and eliminate the disease symptom by exogenous spraying of SA on WT soybean plants (Figure 4), thus establishing a link among *GsCAD1* overexpression, SA elevation and SMV resistance.

### 2.5. GsCAD1-Overexpression Affected the Accumulation of SMV in Soybean Leaves

**Figure 5:**
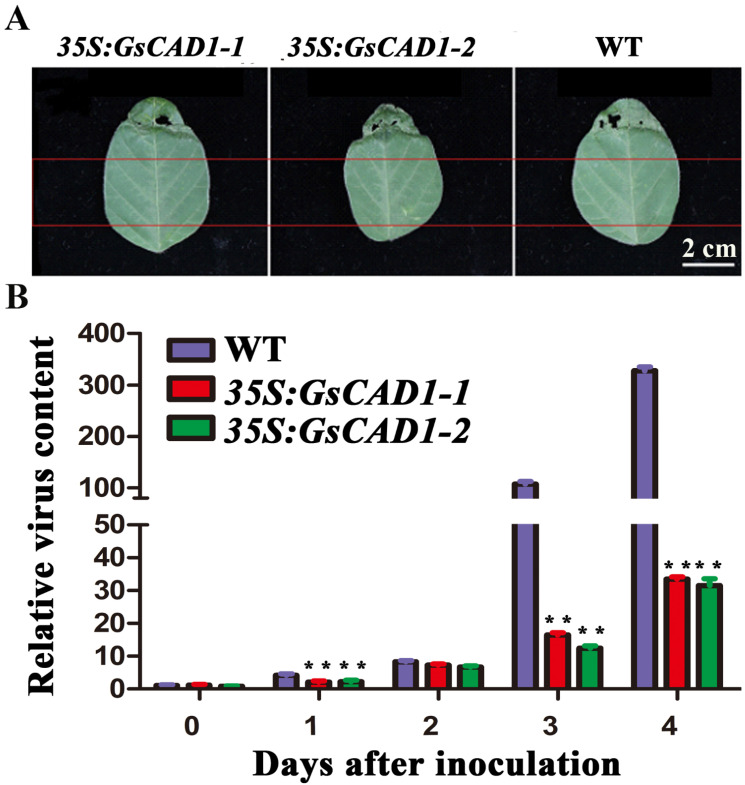
*GsCAD1*-overexpression affected the accumulation of SMV in soybean leaves. (**A**) The first trifoliate leaves of 14-day-old wild-type and *35S:GsCAD1* transgenic plants were inoculated with virus. Leaf sections below the inoculation sites (between the two lines) were collected 1, 2, 3 and 4 days, respectively, after inoculation. Total RNA was isolate and qRT-PCR was used to examine virus RNA. (**B**) Virus content in the wild-type and *35S:GsCAD1* transgenic plants at 1, 2, 3 and 4 days, respectively, after inoculation (*CP* gene). Data represent the mean ± SD of three independent experiments at 35 days of SMV inoculation, with each experiment consisting of three technical replicates. The asterisks in each panel represent significant differences in virus content between WT and OE lines (Student’s *t*-test, **, *p* < 0.01).

The above results have shown that overexpression of *GsCAD1* can significantly boost resistance to SMV. To test if this acquired SMV resistance was related to altered accumulation of the virus in the infested soybean leaves, we inoculated the same part of the fully expanded first pair of three-leaf compound leaves of the transgenic *GsCAD1*-OE lines and non-transgenic WT plants. The samples were then taken from the same part of the inoculated leaves (the upper part) at the same time-points, i.e., 1, 2, 3 and 4 days after inoculation with SMV (Figure 5A), and the virus contents were assessed by qRT-PCR. We found that the SMV concentration in WT plants was significantly higher than those in the *GsCAD1*-OE lines at three time-points, i.e., 1, 3 and 4 days post-inoculation, although no difference was detected at 2 days post-inoculation (Figure 5B). These results suggest that overexpression of *GsCAD1* constrained the accumulation of SMV and thereby enhanced the resistance of the host to the virus.

## 3. Discussion

SMV disease is one of the most devastating diseases of soybeans, which seriously affects the yield and quality of soybean products [47]. In the process of coevolution between SMV and the host, different physiological strains of the virus continue to undergo de novo mutation and recombination to rapidly evolve new and often more virulent strains, resulting in the loss of resistance of the original disease-resistant plants [2,3]. At present, several types of SMV-resistant genes were cloned from resistant varieties or soybean mutant librarys through gene chip, transcriptome sequencing and map-based cloning technologies [3,16,17,19,20,21,24,26,27,29,48]. The cloning of these anti-SMV genes provides molecular targets for more efficient soybean breeding based on molecular design strategy. However, due to the loss of resistance in resistant varieties during planting and the rapid emergence of different physiological strains of SMV [49], it is necessary to clone new resistance genes to cope with the rapid evolution of new SMV strains. China is the center of origin of the cultivated soybean and owns 90% of the world’s wild soybean resources. As the direct progenitor of the cultivated soybean, the annual wild soybean (*G. soja*) has a higher level of genetic diversity, and contains abundant but as yet barely tapped resistance gene resources including those resistant to SMV [41,44,50]. Many wild soybean accessions have been identified as resistant to SMV. For example, of the 129 wild soybean accessions from Hebei province, China, tested, 2.3% showed high and 14.7% showed moderate resistance to various SMVs [51]. Notwithstanding, although the wild and cultivated soybeans can be readily hybridized to form fertile progenies, the ubiquitous linkage drag of unfavorable genes has discouraged soybean breeders from directly using wild soybean resources in their breeding programs [52,53]. Therefore, identifying and cloning new SMV resistance genes from wild soybean resources and transferring them into SMV-susceptible cultivated soybean varieties by the transgenic approach should be a more effective way to improve the resistance to SMV disease in otherwise elite soybean cultivars.

Here, by RNAseq-based transcriptome analysis, we analyzed the gene expression changes of a wild soybean accession BYO-15 with high resistance to SMV and a soybean cultivar Williams 82 that is susceptible to SMV before and after inoculation. We demonstrated that the *GsCAD1* gene was significantly up-regulated and persisted post-SMV-inoculation in BYO-15 and a F3 plant between BYO-15 and Williams 82, R-1, which is also SMV-resistant. In contrast, the *GmCAD1* homolog from Williams 82 did not show this expression pattern in Williams 82 and in an F3 plant, S-1, which is susceptible to SMV (Figure 1A). Notably, there is only a single SNP (T435A) between *GsCAD1* and *GmCAD1*, which resulted in a missense mutation of amino acids (E145D) (Figure 1C). The protein secondary structure was predicted and we found that the existence of this SNP may enable a change from a random coil (GmCAD1) to a β-sheet (GsCAD1) at the 145 amino acid (Figure S3). Thus, our results have clearly shown that overexpression of *GsCAD1* in an otherwise susceptible cultivated soybean confers a high-level of resistance to both of the two contemporarily most virulent SMV strains in China (Figure 2). These results indicate that the construction of *GsCAD1*-OE lines by transgenic approach could improve the resistance to SMV in cultivated soybeans.

The phytohormone SA is known to play an important regulatory role in defense against pathogen infection in plants via inducing hypersensitivity reaction (HR) and/or systemic acquired resistance (SAR) [54]. Many studies have reported that increased SA content can enhance resistance to pathogenic bacteria in plants. In *Arabidopsis thaliana*, increased SA content enhances resistance to turnip mosaic virus [48,55]. In maize, SA accumulation can enhance resistance to sugarcane mosaic virus by limiting virus amplification [48]. In chrysanthemum, *CmWRKY15-1*, via regulating the SA-mediated signaling pathway, enhanced resistance to *P. horiana* infection [56]. In *Nicotiana benthamian*, the down-regulation of SA signaling can promote the intercellular movement of the tobacco mosaic virus [57]. In soybeans, increased SA content greatly contributes to SMV resistance [58]. These prior findings are consistent with our observation in this study that the lignin and SA contents were significantly higher in the SMV-resistant *GsCAD1*-OE lines than in the wild-type after inoculation with the virus. Although the content of SA increased at 3 days after inoculation with SMV in WT plants, the level of increment was lower than that of the *GsCAD1*-OE lines (Figure 3A,B). At the same time, the expression levels of *GmPAL-03g* and *PR1* genes were significantly higher than those of non-transgenic WT plants before and after inoculation with SMV (Figure 3C,D), which was also coincided with the changes in SA content. Notably, we found that the acquired SMV resistance through the overexpression of *GsCAD1* is also related to increased lignin content (Figure 5). Many studies have reported that lignin, acting as the first physical barrier, can withstand the expending of pathogens [32,37,38,39]. There is also a report on the co-regulation of pathogenic bacteria resistance with SA [59]. Our results are consistent with these studies and further confirm that lignin may act as the first physical line of defense against SMV, then SA can activate several plant defense responses, which together constrain either or both the amplification and intercellular movement of SMV. This SMV-resistant working model should be further explored in future investigations.

## 4. Materials and Methods

### 4.1. Plant Materials

Soybean cultivar Williams 82 and a wild soybean accession BYO-15 (wild soybean BYO-15 showed no symptoms of soybean mosaic virus after inoculation with a mixture of SMV strains SC1 and SC3 under both field and greenhouse conditions) and their hybrid descendants in the F3 generation including one resistant plant (R-1) and one susceptible plant (S-1) were used in this study (Figure S1), BYO-15 was picked from Chifeng, Neimenggu Autonomous Region, China. The leaf material before inoculation and 7 and 14 days after inoculation was used for RNA-seq. Susceptible cultivar Williams 82 was used for transformation, resistance analysis and SA treatment.

### 4.2. Virus and SMV Inoculation

A mixture of SMV strains SC1 and SC3, two of the prevalent SMV strains in China, was used for a virus inoculation experiment [20]. Soybean cultivar Williams 82 and wild soybean accession BYO-15 and their hybrid descendants in the S3 generation were grown in a greenhouse with a 16 h/8 h light/dark regime at 25 °C. A mixture of SMV SC1 and SMV SC3 was frozen by liquid nitrogen, then was re-suspended in a 0.01 mol/L phosphate solution at a ratio of 1 g/10 mL. The 14-day-old seedlings with first trifoliate leaves extending were used for inoculation.

### 4.3. Transcriptome Profiling

Leaves were collected before inoculation (0 day), and 7 and 14 days, respectively, after the SMV inoculation (two-week-old seedlings). Total RNA was extracted by TriPure Isolation Reagent (Roche Diagnostics, Mannheim, Germany) according to the manufacturer’s specifications, and used for transcriptome analysis. In addition, RNA sequencing was performed on a HiSeq™ 2500 (San Diego, CA, USA). Low-quality and contaminated sequences were removed from raw data by using the fastp (v0.20.1) [60]. The clean data obtained were aligned to the soybean reference genome (http://www.phytozome.net/soybean, accessed on 12 December 2015) using the TopHat (v2.1.1) software [61]. The gene expression levels were indicated as FPKM (fragments per kilobase of exon model per million mapped reads). Q value ≤ 0.05 and fold change ≥ 2 were considered as significantly different expression genes in two different samples 57. The sequence has been submitted to the NCBI database (BioProject ID: PRJNA898818).

### 4.4. Phylogenetic Analysis of CAD Genes

The full lengths of all the members of the CAD gene superfamily in the soybean and Arabidopsis were aligned by BioEdit. The phylogenetic tree was constructed by using MEGA software (version: 7.0.26.) with neighbor-joining methods (the software was sourced from www.megasoftware.net) [62], The genes are named as *GmCAD1* (*Glyma01g02580*), *GmCAD2* (*Glyma01g02570*), *GmCAD3* (*Glyma18g38670*), *GmCAD4* (*Glyma05g32130*), *GmCAD5* (*Glyma08g15420*), *GmCAD6* (*Glyma08g38430*), *GmCAD7* (*Glyma16g19790*), *GmCAD8* (*Glyma09g33390*), *GmCAD9* (*Glyma14g40170*), *GmCAD10* (*Glyma17g37960*), *GmCAD11* (*Glyma15g06460*), *GmCAD12* (*Glyma20g26440*), *GmCAD13* (*Glyma10g40870*), *GmCAD14* (*Glyma13g32830*), *GmCAD15* (*Glyma07g28040*) and *GmCAD16* (*Glyma08g37510*).

### 4.5. Gene Clone and Vector Construction

The new trifoliate leaves were collected at 0, 7 and 14 days after inoculation with the SMV mixture, respectively. Total RNA was extracted by TriPure Isolation Reagent (Roche Diagnostics, Mannheim, Germany) according to the manufacturer’s specifications. One μg RNA of each sample was reverse-transcribed to cDNA by a reverse transcription kit (Transgen Biotech, Beijing, China) according to the manufacturer’s protocol. The *GsCAD1* (*Glyma01g02580*) and *GmCAD1* genes were amplified by PCR with forward primer 5′-CTCTAGAATGGCAGCACAAGCTGAA-3′ and reverse primer 5′-CGGATCCGAATTTCAGTGTGTTTCCA-3′. The amplified DNA fragments were inserted into vector *pTF101.1-35S* by incision and ligation with endonucleases *Xba*I, *Bam*HI and T4 DNA ligase (NEB, Ipswich, MA, USA).

### 4.6. Soybean Transformation and Screening of Transgenic Plants

Constructed plasmids were transformed into cultivar Williams 82 by *Agrobacterium-tumefaciens*-mediated cotyledonary node transformation [63]. We screened T2 transgenic plants by 2‰ Basta. We further confirmed the surviving plants after Basta treatment by a PCR-detected phosphinothricin acetyltransferase selectable marker gene (BAR) with forward primer 5′-ACTATCCTTCGCAAGACCCTT-3′ and reverse primer 5′-ACTCTAATCATAAAAACCCATCTCA-3′. Quantitative RT-PCR (qRT-PCR) was used to examine the expression levels of *GsCAD1*, while *GmActin11* was used as an internal control.

### 4.7. Quantitative RT-PCR (qRT-PCR)

Total RNA from different tissues of the transgenic plants and wild-type (WT) plants were isolated by using the EasyPure Plant RNA Kit (Transgen Biotech, Beijing, China). The cDNA was synthesized using the ThermoScript RT-PCR system (Invitrogen, Carlsbad, CA, USA), then 1 µL of the diluted cDNA was used for qRT-PCR. The qRT-PCR conditions were: 50 °C for 2 min, 95 °C for 10 min, and 35 cycles of 95 °C for 2 min, 60 °C for 30 s and 72 °C for 30 s. The qPCR analyses that were performed with each sample had three independent experiments, as each experiment had three technical replicates. All of the qRT-PCR primers used are listed in Supplemental Table S9.

### 4.8. SMV Resistance Assay

Two T3 transgenic lines, *35S:GsCAD1-1* and *35S:GsCAD1-2*, together with WT Williams 82, were grown in a greenhouse at 25 °C with a 16 h/8 h light/dark regime. Twenty seedlings were selected for each transgenic line, 10 two-week-old seedlings from each line were inoculated with SMV, and another five seedlings from each line were inoculated with a 0.01 mol/L phosphate solution as a control treatment. The remaining five seedlings from each line were used for leaf inoculation to analyze the speed of SMV transmission. We wounded the upper part of the leaf by rubbing SMV inoculums, and abandoned the inoculation sites, and the woundless half-bottoms of the leaves, before inoculation (0 h), and 72 h after inoculation, were collected. The woundless segments were used for RNA extraction, and then to measure SMV content by qRT-PCR.

### 4.9. Salicylic Acid Treatment

Fourteen-day-old seedlings of WT were sprinkled with 0.1 g/L salicylic acid (SA) as the experimental group and plants treated with distilled water were used as the mock control group, then both groups of plants were inoculated with SMV as described above. Half-bottoms of leaves from both groups 3 days after inoculation were collected. The relative content of SMV was detected by qRT-PCR.

### 4.10. Measurement of SA and Lignin Contents

Lignin content was measured by a biochemic kit (Solarbio, Beijing, China). The content of dissociative SA was measured by High Performance Liquid Chromatography (HPLC) in Keming Biotechnology Co., Ltd. (Suzhou, China). Transgenic lines *35S:GsCAD1-1* and *35S:GsCAD1-2,* along with WT, were inoculated against SMV. Corresponding leaves were collected before inoculation (0 day) and 3 days after inoculation. The collected leaves were sent to the company above to measure SA and lignin, and each sample had five biological duplicates. Seven SA synthesis genes, *GmPAL* isoforms *Glyma02g47940* (*GmPAL-02g*), *Glyma03g33880* (*GmPAL-03g*), *Glyma10g06600* (*GmPAL-10g*), *Glyma13g20800* (*GmPAL-13g*) and *Glyma19g36620* (*GmPAL-19g*), and *GmICS* isoforms *Glyma01g25690* (*GmICSa*) and *Glyma03g17420* (*GmICSb*), along with pathogenesis-related 1 (*PR1*) (AI930866), were selected for qRT-PCR validation [51], and the expression of *ACT11* (BW652479) was used as an internal control for normalization with forward primer 5′-ATCTTGACTGAGCGTGGTTATTCC-3′ and reverse primer 5′-GCTGGTCCTGGCTGTCTCC-3′, as reported [17].

## Data Availability

All the datasets used and/or analyzed throughout this study can be available from the corresponding authors on reasonable request.

## Supplementary Materials

The following supporting information can be downloaded at: https://www.mdpi.com/article/10.3390/ijms232315206/s1, Figure S1: Flow chart of cloning the anti-SMV genes from the wild soybean; Figure S2: Screening of candidate resistance genes; Figure S3: Prediction of CAD1 protein secondary structure; Table S1: List of gene expression levels were indicated as FPKM (BYO-15-0D-vs-7D); Table S2: List of gene expression levels were indicated as FPKM (BYO-15-0D-vs-14D); Table S3: List of gene expression levels were indicated as FPKM (Ws82-0D-vs-7D); Table S4: List of gene expression levels were indicated as FPKM (Ws82-0D-vs-14D); Table S5: List of gene expression levels were indicated as FPKM (R-1-0D-vs-7D); Table S6: List of gene expression levels were indicated as FPKM (R-1-0D-vs-14D); Table S7: List of gene expression levels were indicated as FPKM (S-1-0D-vs-7D); Table S8: List of gene expression levels were indicated as FPKM (S-1-0D-vs-14D); Table S9: Primers for qRT-PCR.
