# Supplementary material for: Overexpression of a Cinnamyl Alcohol Dehydrogenase-Coding Gene, GsCAD1, from Wild Soybean Enhances Resistance to Soybean Mosaic Virus
Source: Int J Mol Sci. 2022 Dec 2;23(23):15206. doi: 10.3390/ijms232315206 (PMC9740156; doi:10.3390/ijms232315206)
Supplement: Supplementary file 1 [file ijms-23-15206-s001.zip › ijms-2026041-supplementary.pdf]

Overexpression of a Cinnamyl Alcohol Dehydrogenase-Coding Gene, *GsCAD1*, from Wild Soybean Enhances Resistance to Soybean Mosaic Virus

Hongwei Xun [1,2,†], Xueyan Qian [2,†], Meng Wang [1], Jiaxin Yu [1], Xue Zhang [1], Jinsong Pang [1], Shucai Wang [1], Lili Jiang [1,*], Yingshan Dong [2,*] and Bao Liu [1]

[1] Key Laboratory of Molecular Epigenetics of MOE, Northeast Normal University, Changchun 130024, China
[2] Jilin Provincial Key Laboratory of Agricultural Biotechnology, Jilin Academy of Agricultural Sciences, Changchun 130033, China
* Correspondence: jiangll269@nenu.edu.cn (L.J.); ysdong@cjaas.com (Y.D.)
† These authors contributed equally to this work.

This file includes:
Figure S1 to S3

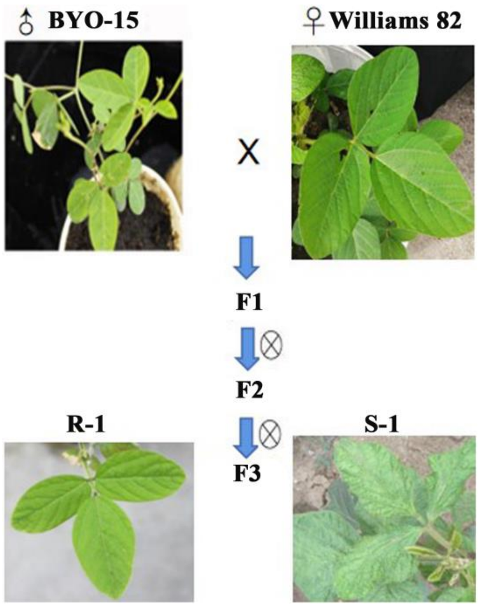

Figure S1. Flow chart of cloning the anti-SMV genes from the wild soybean.

The hybrid of Williams 82 and wild soybean accession BYO-15 produced a resistant (R-1) and a susceptible plant (S-1) after two successive generations of selfing. The phenotypes of all types of plants are at one month stage after SMV inoculation.

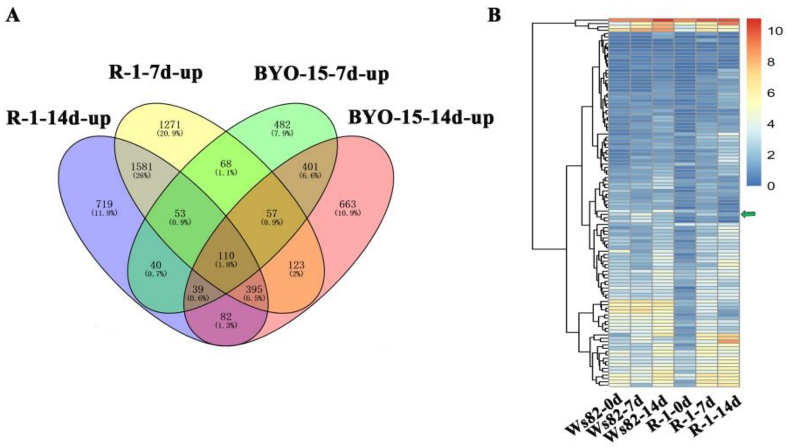

Figure S2. Screening of candidate resistance genes.

The Venny diagram of up-regulated genes (log2 (fold change) ≥ 2) in different plant types of RNA-Seq including wild soybean (BYO-15) and R-1 (7d and 14d after SMV induction). B. The expression level of 110 candidate genes in Williams 82 and S-1 (0d, 7d and 14d before and after SMV induction). The green arrow represents *GmCAD1*. The bar on the right represents log2(RPKM+1).

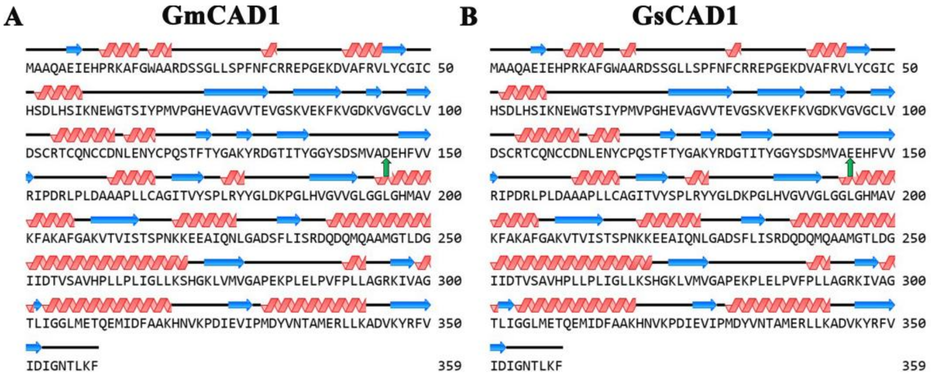

Figure S3. Prediction of CAD1 protein secondary structure.

The protein secondary structure of GmCAD1. **B.** The protein secondary structure of GsCAD1. The green arrow represents the random coil (GmCAD1) was changed to β-sheet (GsCAD1) at the 145 amino acid.
