# Supplementary material for: Overexpression of a Cinnamyl Alcohol Dehydrogenase-Coding Gene, GsCAD1, from Wild Soybean Enhances Resistance to Soybean Mosaic Virus
Source: Int J Mol Sci. 2022 Dec 2;23(23):15206. doi: 10.3390/ijms232315206 (PMC9740156; doi:10.3390/ijms232315206)
Supplement: Supplementary file 1 [file ijms-23-15206-s001.zip › Table S9. Primers for qRT-PCR.pdf]

Table S9. Primers for qRT-PCR

Primers	Sequences
GmActin11-F	ATCTTGACTGAGCGTGGTTATTCC
GmActin11-R	GCTGGTCCTGGCTGTCTCC
SMV-CP-F	AACAGGGCAAGGGAAGCAAT
SMV-CP-R	CCATGCCCAAAAGAGTGTGC
GmPAL-02G-F	GGATAATGCACGTTTAGCTGTT
GmPAL-02G-R	CTTCGACAGTTTTCAAAGCAGA
GmPAL-03G-F	ACTGAATCAAGCCATACCCTG
GmPAL-03G-R	TGTTCAGGAGCTTGGTGATG
GmPAL-10G-F	ACTTCAATATTTGGCGAATCCG
GmPAL-10G-R	AGTCCTCTTAGCAACTTGACTC
GmPAL-13G-F	TTACTCTCCTCGATTTTACCGC
GmPAL-13G-R	ATGTAGCCGTTGTTGTTAACAC
GmICS-01G-F	TCCATCAACTGATACGTTTTGC
GmICS-01G-R	AAGCCAGTAGATGTCATGCTAA
GmICS-03G-F	ATGGGTTAAATCAAGGTGCTTG
GmICS-03G-R	AAACAAAATACTGCATCGGTGG
GmPR1-F	GTGGGTGTTCAAAACTTGGC
GmPR1-R	AGCAAGATTCTCCCCGTATTG
GmCAD1-F	GAGGTGGGAAGCAAAGTAGAG
GmCAD1-R	ACCATATGTGAACGTAGATTGAGG
